# Editorial: Achieving impacts at scale in early childhood interventions: innovations in monitoring, evaluation, and learning

**DOI:** 10.3389/fpubh.2025.1604737

**Published:** 2025-05-09

**Authors:** Abhay Gaidhane, Jane Fisher, Wiedaad Slemming, James Radner, Mahalaqua Nazli Khatib, Penny Holding, Zahiruddin Quazi Syed

**Affiliations:** ^1^Jawaharlal Nehru Medical College, Datta Meghe Institute of Higher Education and Research, Wardha, India; ^2^Jean Hailes Research Unit, School of Public Health and Preventive Medicine, Monash University, Melbourne, VIC, Australia; ^3^Faculty of Health Sciences, Children's Institute, University of Cape Town, Cape Town, South Africa; ^4^Munk School of Global Affairs & Public Policy, University of Toronto, Toronto, ON, Canada

**Keywords:** early childhood development (ECD), measurement for change, monitoring evaluation and learning, impact at scale, improved implementation

In this paper, we share the experience of the editors of two linked series that explore learning from interventions in early child development (ECD) (1). The series encouraged discussion on intentional information use and its embedding in developing sustainable systems. Authors were asked to reflect on how information can do more than just record the past by helping re-frame the future. As we intended to explore innovation and shifts in thinking, we reflect on the learning shared and the structures and systems that have supported or hindered the process.

Measurement for Change (M4C) [(2, 3); de Laat et al.], the framework of information use that stimulated these series, provokes intentional reflection on effective delivery and sustainable scaling. While both series were open calls to those working in implementation, the launch of each series was preceded by workshops for invited authors that outlined the framework. The series coordinators who facilitated the workshops were a different group from ours, the editorial team.

Authors from both series have made a palpable contribution to bringing diverse perspectives into the discussion, while also raising the importance of the analysis of process. Overall, we conclude that the principles proposed by M4C have yet to be sufficiently explored by the whole implementation ecosystem, and that the journey to decolonized thinking requires more intentional sharing of information garnered across multiple contexts.

*1*. ***Effective delivery**: given that the initial intention was to highlight how people get things done, not just what they do, what have the two series contributed to raising the importance of process?*

Our reflections, informed by the critical appraisal of the narratives as well as direct experience of implementing interventions, identify that while vulnerabilities appear from the planning stage of an intervention, responses are often delayed by a lack of awareness of what is missing and the over-concentration in information systems on measuring what has been achieved. The reports in these series were distinctive in that they provided considerable detail about the monitoring, evaluation and learning (MEL) of process, a welcome opportunity given the traditional pressure to focus on results, on measurement *of* change (Watkins et al.; Nair et al.). This exploration aligns with the rapid evolution of the discussion on decolonisation that has been unfolding across the implementation ecosystem since the launch of the first series.

While the language of M4C was not generally applied in these narratives, the interest in the series suggests a hunger for the exploration of the whole implementation system, demonstrating what can happen when a different lens is used. However, the papers illustrate that co-design, and the dismantling of hierarchy in decision making, which we regard as an essential component of sustainable impact, has yet to become centralized in the dialogue and in practice.

*2*. ***Transition to scale**: what useful themes have emerged regarding how the challenge of sustainable scaling can/has been met?*

Population-level ECD implementation involves embedding change in government structures and enabling population-level engagement, ownership, and cultural change (Volen). This requires attention to a broader narrative, one that captures the complexity of systems and human behavior (Murdock et al.).

However, many narratives included in the series address scaling as an increase in the numbers rather than the embedding of interventions into local systems, or the systematic removal of inequality. Success was generally simplified as transfer to government, with few narratives detailing failure, which is actually more common. Lacking, too, are narratives on developing a long game: on the collective building of the resources required for scale up and of methods to sustain value systems over time.

The invitation to reflect on what scaling means, and what is effective in creating sustainability, was fully taken up by only a few authors (Shaw and da Silva). Narratives identified the value of creating loops of information (Brien et al.), but the description of implementation processes commonly remained linear, with the complexity of the networks and systems implicated in building sustainability and scale remaining largely unexplored (Vohra et al.).

*3*. ***Changing the narrative**: potential authors raised concerns that it would be difficult to publish narratives on process. To what extent were their concerns validated, and/or overcome?*

The level of response to the call for this series reflects a real need for a public forum, particularly on process. The first series on effective delivery comprised 32 papers, produced by 157 authors (Vohra et al.), and the second, focused on scaling, included 20 papers, and 82 authors. Indeed, implementation science has legitimized engagement with process, providing multiple frameworks and theories, some of which were used by series authors (Sklar and Murokora). More prescriptive frameworks may be more readily mirrored in the structure of writing, but what is illustrated across these papers was the importance of reflective practice that is central to M4C. The intention to think deeply transformed data from information into learning (Nair et al.).

M4C also champions continuous evolution of thinking (Gaidhane et al.), and this is mirrored in the shifts seen between the two series (Apte and Pahan). The inclusion of new voices was a major achievement. For many authors, writing for peer reviewed journals was a new experience. In the second series new voices brought in perspectives from outside the early childhood field, opening up a broader spectrum of ideas and contexts (Krause et al.). This aligns with M4C's goal of ecosystem change (Landon et al.), where all programme participants, notably including funders, engage with a broader range of stakeholders, and apply a human rights based approach to programme design.

While there is a ground swell moving away from centering decision making on randomized control trials alone, many papers still focused on effect size, reporting on achievements without also exploring how change and progress occurred. This is to be expected while authors struggle with the traditional format commonly outlined by publishers. Departures from the standard sequence—Introduction, Methods, Results, and Discussion—also acted as a constraint on generating a pool of reviewers willing to critique narratives centered on process and not on outcome.

Reviewers who were adequately primed on the intention of the series actively engaged with narratives that included speculative commentary. Within this approach reviewers sought revisions that increased the transparency of the reflective process. Demanded from authors was a level of rigor in identifying the speculative, and detailing of the information that triggered the speculation.

*4*. ***Changing the paradigm**: the intention of M4C has been to ensure that design, evaluation and planning are diverse, equitable, and inclusive. What have been the successes observed, and what remain the key challenges in decolonizing implementation science?*

The two series reflect a measure of success in the decolonization of thought (Watkins et al.; Rasheed), with authors looking from a more holistic perspective and questioning linear frameworks in the decision making process (Dusabe et al.). However, boundaries need to be pushed further. We need explanations of the scaling process that are informed by clearly structured intentional MEL frameworks, and that expand understanding of variability and complex systems (Anago et al.).

Diversity of voices is important to challenge and change, and we were able to draw in multiple voices. Together the papers open a window to how different cultures and communities use evidence, recognize specific gaps, and generate a collaborative planning and consultation process [Shaw and da Silva; (4)]. Nonetheless we still witnessed a dependency on the values and principles of high-income northern partners both in the choice of content shared and in how narratives were structured. Implementation science and the donor community remain overly tied to values that originate in the global north, while decolonisation depends upon being deeply informed by context and variability.

The support shown by Frontiers, inviting us back for a second series, has been a pivotal part of the process of changing the discussion. To continue the dialogue more constructively, publishing culture must also change, encouraging narratives that expand beyond impact to explore features of replication, sustainability, and scale. Journals must actively seek narratives that speak to wider audiences, in different languages.

For many countries, resources targeted at ECD still lag behind those focused on survival. To bridge this gap a parenting product is often imposed, rather than evolving out of homegrown methods and solutions already embedded in local goals. Funding structures too must therefore change to support a broader capacity to grow alternatives and embed delivery.

*5. M4C suggests* ***five key principles are*** *implicated in effective practice and sustainable scaling:* ***Dynamic, Inclusive, Informative, Interactive, People Centered.*** *What examples have you seen included in the narratives shared?*

While most contributions did not name the five M4C principles explicitly, their intention was implicit in many of the narratives. A fixed narrative structure based on M4C was not prescribed by the series co-ordinators, so it is hard to draw firm conclusions on the absence of the language of M4C, or the value of the five principles for different authors.

In almost all the narratives information was used in a generative way. A Dynamic, iterative, and Interactive approach to implementation design that moves away from fixed protocols featured in many narratives. Implementation teams did not necessarily wait for a final report of results to make modifications and improve practice, although descriptions of fully Informative and regular data sharing across a broad network of stakeholders were uncommon (Coore-Hall et al.).

Understanding and responding to variability in needs, being People Centered, also featured, although generally this principle was narrowly applied, and multiple levels of meaning were not explored systematically. As we have already shared, actively building long term collaborations, part of being Inclusive, was even less commonly described.

6. ***Reflections on what we learnt about information use***

The means and methods to embed an information system in a programme, through inclusive tools, methods, or collective decision making, were variously explored in the narratives. Implementation teams generally used a variety of data, and looked beyond the numbers (López et al.). Still, the quantitative tradition dominated, with only a few contributions describing application of more innovative methods to capture contextual meaning and or deepen understanding through triangulation across means and Slemming et al. (5). Another obvious gap was the lack of explorations of consequences, particularly the unanticipated. As the narratives from the COVID-19 pandemic illustrate, response to the unanticipated can provide a powerful source of learning on resilience and adaptability.

There appears to remain a lack of confidence that novel information tools and methods will be accepted by academia (publishers and reviewers), and by gatekeepers (funders and global organizations). Consequently, a rich opportunity to explore alternative frameworks for design and implementation, and to develop rigorous and systematic use of stories, especially those that learn from the missed and from failure, remains untapped.

*7. Reflections on what we* ***experienced as editors,*** *what helped and what hindered us getting this series completed*.

An irony in M4C is that, in its championing of de-colonized thought, it strives not to be prescriptive of means and methods. This has made it difficult to carry out a systematic review of each of the five proposed principles. But as the intention was not to introduce a framework, but to change practice, in our failure to achieve outcome—use of M4C language—we also see success - in the achievement of a broad range of detailed narratives on changed perspectives and good practice. Future initiatives could gain greater insight through the application of a more structured invitation for practitioners to actively use the M4C framework in their reflective practice, with a systematic guide for potential authors and reviewers to apply in their critical appraisal of the learning.

The starting point for any paradigm shift is to challenge personal frameworks and structures. The conceptualization of what constitutes evidence has shifted for those involved in the production of these series, particularly toward acknowledging engagement in learning as crucial to change. The papers have made explicit the value of an inclusive process for gathering and interpreting information and for considering who is listening. Implicit is the need to create a dynamic, though structured, process to ignite real and lasting change.
